# A Case of Conjunctival Cyst Required Removal Six Months After Strabismus Surgery

**DOI:** 10.7759/cureus.66433

**Published:** 2024-08-08

**Authors:** Yohei Takahashi, Toshiaki Goseki, Sonoko Tatsui, Hitoshi Ishikawa, Nobuyuki Shoji

**Affiliations:** 1 Department of Ophthalmology, Tokyo Women’s Medical University, Tokyo, JPN; 2 Department of Ophthalmology, Kitasato University School of Medicine, Kanagawa, JPN; 3 Department of Ophthalmology, International University of Health and Welfare Atami Hospital, Shizuoka, JPN; 4 Department of Orthoptics and Visual Science, Kitasato University School of Allied Health Sciences, Kanagawa, JPN

**Keywords:** conjunctival epithelium, lateral rectus recession surgery, histopathological examination, postoperative complication, conjunctival cyst

## Abstract

A conjunctival cyst is a rare yet significant complication following strabismus surgery. This report describes a nine-year-old girl who developed a conjunctival cyst after undergoing bilateral lateral rectus recession surgery for intermittent exotropia. Despite an uneventful surgery and standard postoperative care, she presented with a gradually enlarging subconjunctival mass in the left eye three months later. Initial conservative treatment with topical antibiotics and steroids proved ineffective, leading to surgical excision six months postoperatively. Histopathological examination confirmed the cyst as a conjunctival epithelial inclusion cyst, characterized by cuboidal epithelium containing goblet cells. The patient’s postoperative course was uneventful, with no recurrence of the cyst at six months follow-up, stable visual acuity, and maintained strabismus correction. In managing this case, two crucial lessons were learned. Firstly, the need for precise surgical techniques and the use of adequate pre- and intraoperative disinfection measures to prevent postoperative complications. Ensuring that the conjunctival tissue is not inadvertently included in the wound closure and maintaining a sterile environment throughout the surgery are critical steps. Secondly, the importance of early recognition and timely intervention for postoperative complications. The patient’s cyst developed three months post-surgery and did not respond to conservative treatments, necessitating surgical excision. This reinforces the need for heightened awareness and prompt surgical intervention when conservative measures fail, ensuring optimal patient outcomes and avoiding unnecessary discomfort or cosmetic issues. In conclusion, meticulous surgical technique and proper pre- and intraoperative disinfection are paramount in preventing postoperative complications such as conjunctival cysts. Early recognition and timely surgical intervention are essential for managing these cysts effectively. This case reinforces the importance for ophthalmic surgeons to remain vigilant in their surgical practices and to promptly address any postoperative complications, thereby improving surgical outcomes and enhancing patient care in strabismus surgery.

## Introduction

Conjunctival cysts are infrequent yet significant complications that can arise following strabismus surgery, with an incidence ranging from 0.25% to 2.3% in various studies [1-3]. These cysts can result from several etiologies, including infections, suture allergies, and the inadvertent inclusion of conjunctival epithelium during the surgical procedure [3]. Although often benign, conjunctival cysts can necessitate surgical intervention when conservative treatments, such as topical medications, prove ineffective [3-5].

This case report details the development and management of a conjunctival cyst in a nine-year-old girl who underwent strabismus surgery for intermittent exotropia. The patient's post-operative course was complicated by the formation of a conjunctival cyst, a rare but notable occurrence. Given the complexity and rarity of this complication, it is imperative to understand the underlying mechanisms and effective treatment modalities to improve surgical outcomes and patient care.

The patient presented with a gradually enlarging subconjunctival mass in the left eye, which did not resolve with standard medical therapy. Surgical excision was ultimately required due to the persistence and size of the cyst. Histopathological examination confirmed the diagnosis of a conjunctival epithelial inclusion cyst, characterized by a cystic structure lined with cuboidal epithelium containing goblet cells.

There are several reports on the development of conjunctival cysts, including the pathological findings [2-5], but there have been few detailed studies [6,7]. This report highlights the importance of appropriate surgical techniques and adequate pre- and intraoperative disinfection to prevent the inclusion of conjunctival epithelium and subsequent cyst formation. Furthermore, it underscores the necessity of considering this rare complication in the differential diagnosis of post-strabismus surgery patients presenting with conjunctival masses. Early recognition and appropriate management are crucial in preventing further complications and ensuring optimal visual and functional outcomes for the patient.

## Case presentation

A nine-year-old girl presented with intermittent exotropia, which had progressively worsened over the past year. The patient had no significant past medical history, allergies, or previous ocular surgeries. After a thorough ophthalmologic evaluation, bilateral lateral rectus recession surgery was recommended to correct the strabismus.

The patient underwent bilateral lateral rectus recession surgery under general anesthesia. The procedure was uneventful, and standard surgical techniques were employed. Conjunctival incisions were made, and the lateral rectus muscles were identified, dissected, and reattached at the predetermined positions. Conjunctival closure was performed using 7-0 polyglactin sutures. No intraoperative complications were noted.

The immediate postoperative course was unremarkable, and the patient was prescribed a regimen of antibiotic (levofloxacin 1.5%) and steroid (fluorometholone 0.1%) eye drops. At the one-week follow-up, the surgical sites appeared to be healing well, with no signs of infection or inflammation. The patient reported mild discomfort, which was managed with analgesics.

**Figure 1:**
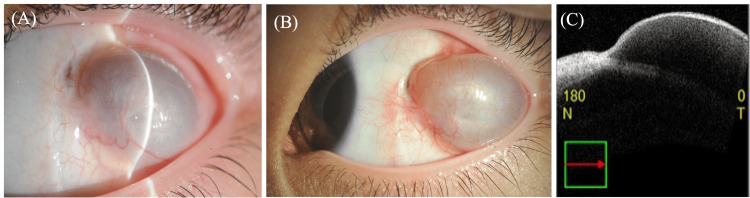
Images of the cyst at three and six months post-operatively, and anterior segment OCT images at three months post-operatively. (A) Three months postoperatively, a subconjunctival cystic lesion appeared in the left ocular auricle. (B) Six months postoperatively, the lesion showed a gradual tendency to enlarge. (C) Anterior ocular optical coherence tomography (OCT) performed three months postoperatively showed a subconjunctival cystic lesion, which was unifocal and showed internal hyporeflexia.

The appearance of a cyst was observed one month after surgery and the patient was aware of it. Initially, the cysts were not very large and were followed up with continuous eye drops. Approximately three months postoperatively, the patient returned with complaints of a gradually enlarging mass in the left eye. On examination, a subconjunctival cystic lesion was observed in the temporal conjunctiva. The cyst was translucent, dome-shaped, and measured approximately 10 x 8 mm. There were no signs of infection, such as redness, discharge, or pain. Anterior segment optical coherence tomography (OCT) confirmed the presence of a cystic structure containing clear fluid (Figure 1).

Initial management included conservative measures with continued use of topical antibiotics (levofloxacin 1.5%) and steroids (fluorometholone 0.1%). However, after three months of follow-up, the cyst showed no signs of regression and continued to increase in size, causing discomfort and cosmetic concern. At this point, surgical excision was considered necessary.

**Figure 2:**
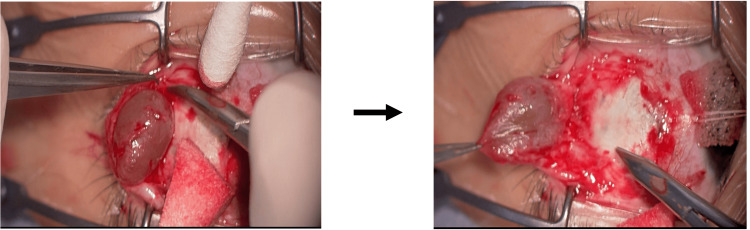
Intraoperative images The cyst adherent to the sclera was sharply dissected and completely removed without rupturing it.

The patient underwent surgery for excision of the conjunctival cyst. Under general anesthesia, a conjunctival incision was made over the cyst. The cyst wall was carefully dissected from the surrounding tissue and excised in toto. Intraoperative findings revealed a cyst firmly adherent to the underlying sclera, necessitating sharp dissection (Figure 2). Meticulous technique was employed to ensure the complete removal of the cyst without rupture.

**Figure 3:**
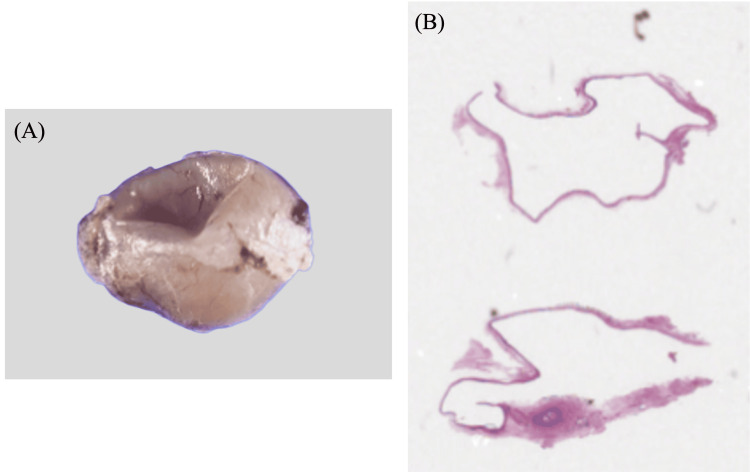
Pathological images (A) Macroscopically, the cyst measured 10 x 8 x 6 mm and contained clear serous fluid. (B) Microscopically, the cyst was lined by cuboidal epithelium with goblet cells, consistent with a conjunctival epithelial inclusion cyst.

The excised cyst was sent for histopathological examination. Macroscopically, the cyst measured 10 x 8 x 6 mm and contained clear serous fluid. Microscopically, the cyst was lined by cuboidal epithelium with goblet cells, consistent with a conjunctival epithelial inclusion cyst (Figure 3). No inflammatory or granulomatous changes or malignant findings were observed.

**Figure 4:**
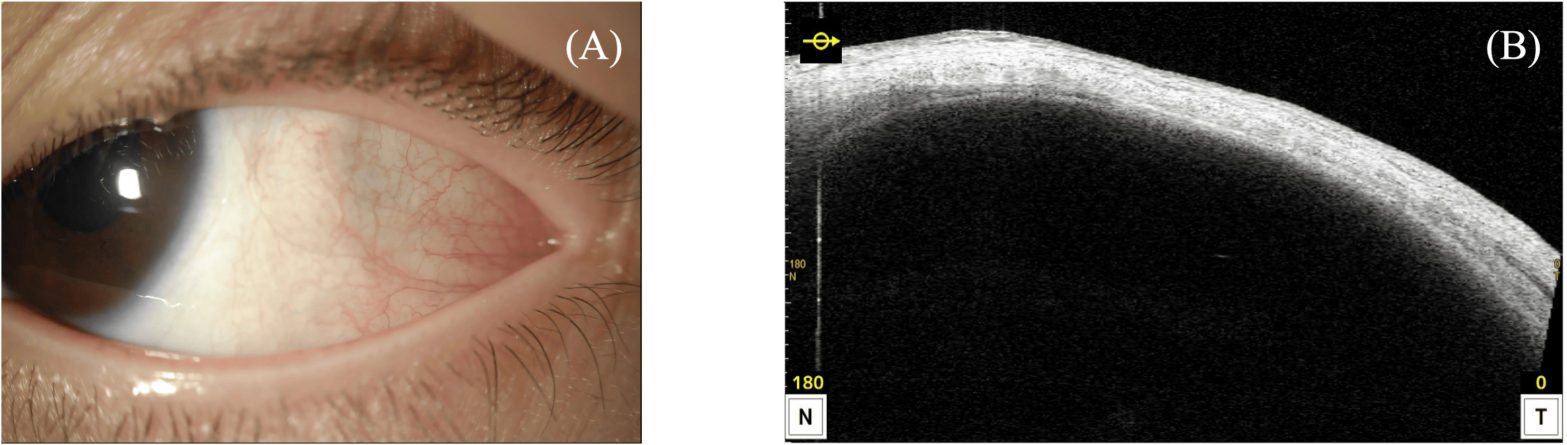
Postoperative image of the conjunctiva and anterior segment OCT image (A) Conjunctival images (B) Anterior segment optical coherence tomography (OCT) image No recurrence of cysts on either conjunctival or anterior segment images up to six months postoperative follow-up.

The postoperative course was uneventful, with no recurrence of the cyst noted at follow-up visits up to six months after surgery (Figure 4). The patient’s visual acuity remained stable, and the strabismus correction was maintained. The conjunctival incision healed well without scarring or signs of infection.

## Discussion

In this case, we learned two crucial lessons in managing complications arising from strabismus surgery. Firstly, meticulous surgical technique and proper pre- and intraoperative disinfection are paramount in preventing postoperative complications such as conjunctival cysts. Secondly, recognizing and addressing conjunctival cysts promptly is essential to ensure optimal patient outcomes and avoid unnecessary discomfort or cosmetic issues.

In previous reports, conjunctival cyst formation post-strabismus surgery has been attributed to several factors, with the inclusion of conjunctival epithelium being the most common [3]. This case highlights the importance of precise surgical techniques and the use of adequate pre- and intraoperative disinfection measures. During the surgery, careful handling of conjunctival tissues and the use of iodine solution to cleanse the surgical field can significantly reduce the risk of epithelial inclusion and subsequent cyst formation [2,3]. On the other hand, allergy to sutures has also been reported as another factor in postoperative conjunctival cyst formation [3]. In this case, since no cyst formation was observed in the fellow eye using the same suture as the affected eye, it was considered unlikely that suture allergy was the cause. Furthermore, the length of surgery time has been cited as a factor in the formation of conjunctival cysts after strabismus surgery [8]. In fact, in the present case, the operative time of the eye in which the cyst developed was longer than that of the opposite eye. Our patient's development of a conjunctival cyst, despite standard postoperative care, underscores the need for heightened awareness and diligence in surgical procedures. Ensuring that the conjunctival tissue is not inadvertently included in the wound closure, maintaining a sterile environment throughout the surgery, and keeping the operative time as short as possible are critical steps in preventing such complications.

Secondly, early recognition and timely intervention are crucial when postoperative complications like conjunctival cysts arise. In this case, the cyst developed three months after surgery and did not respond to conservative treatment with topical antibiotics and steroids. The persistence and gradual enlargement of the cyst necessitated surgical excision. This case underscores the importance of considering conjunctival cysts in the differential diagnosis of postoperative conjunctival lesions. When conservative treatments fail, prompt surgical intervention is required to alleviate symptoms and prevent further complications [3-5]. The excision of the cyst in our patient resulted in a successful outcome, with no recurrence noted at follow-up visits. This highlights the effectiveness of surgical excision in managing conjunctival cysts and the necessity of considering this option when conservative measures are insufficient.

## Conclusions

In conclusion, meticulous surgical technique and proper pre- and intraoperative disinfection are critical in preventing postoperative complications such as conjunctival cysts. Furthermore, early recognition and timely surgical intervention are essential in managing these cysts effectively. This case reinforces the need for ophthalmic surgeons to be vigilant in their surgical practices and to promptly address any complications that may arise postoperatively. By adhering to these lessons, we can improve surgical outcomes and enhance patient care in strabismus surgery.
